# An investigation into the associations between psychological skills, anaerobic fitness, and aerobic fitness in elite Iranian taekwondo athletes

**DOI:** 10.1371/journal.pone.0288227

**Published:** 2023-07-07

**Authors:** Maghsoud Nabilpour, Mohammad Hossein Samanipour, Nicola Luigi Bragazzi, Monoem Haddad, Tomás Herrera-Valenzuela, Dan Tao, Julien S. Baker, Jožef Šimenko

**Affiliations:** 1 Department of Sports Physiology, Faculty of Educational Sciences and Psychology, Mohaghegh Ardabili University, Ardabil, Iran; 2 Department of Sport Science; Imam Khomeini International University, Qazvin, Iran; 3 Laboratory for Industrial and Applied Mathematics (LIAM), Department of Mathematics and Statistics, York University, Toronto, Ontario, Canada; 4 Physical Education Department, College of Education, Qatar University, Doha, Qatar; 5 Department of Physical Activity, Sports and Health Sciences, Faculty of Medical Sciences, Universidad de Santiago de Chile (USACH), Santiago, Chile; 6 Department of Government and International Studies, Hong Kong Baptist University, Hong Kong, China; 7 Center for Health and Exercise Science Research, Department of Sport, Physical Education and Health, Hong Kong Baptist University, Hong Kong, China; 8 School of Life and Medical Sciences, University of Hertfordshire, Hatfield, United Kingdom; University of Study of Bari Aldo Moro, ITALY

## Abstract

This study investigated the relationship between psychological skills and fitness levels among elite taekwondo athletes. A total of ten Iranian male elite taekwondo athletes (mean age of 20.6±2 years, BMI 18.78±0.62 kg/m^2^, and fat percentage of 8.87±1.46%) participated in the study. The Sports Emotional Intelligence Questionnaire, Sports Success Scale, Sport Mental Toughness Questionnaire, and Mindfulness Inventory for Sport were used to assess psychological factors. The Wingate test was used to determine anaerobic power, and the Bruce test to determine aerobic fitness. Descriptive statistics and Spearman rank correlation coefficients were utilised to examine any relationships between subscales. Statistically significant correlations were recorded between the evaluation of feelings (EI scale) and VO_2_peak (ml/kg/min) (r = -0.70, p = 0.0235) and between social skills (EI scale) and relative peak power (W/kg) (r = 0.84, p = 0.0026). Also, between optimism (EI scale) and VO_2_peak (ml/kg/min) (r = -0.70, p = 0.0252) and between optimism (EI scale) and HR-MAX (r = -0.75, p = 0.0123); and, finally, between control (mental toughness scale) and relative peak power (W/kg) (r = 0.67, p = 0.0360). These findings demonstrate relationships between psychological factors and the advantages of good anaerobic and aerobic capabilities. Finally, the study also demonstrated that elite taekwondo athletes have high mental performance abilities that are interrelated with anaerobic and aerobic performance.

## 1. Introduction

Sports psychologists suggest that the human psyche is directly affected by physical fitness, and, reciprocally, human physical performance is influenced by an athlete’s psychological and mental attitude [1]. Accordingly, researchers in sports psychology have concluded that the more athletes can understand, identify and regulate the precise expression of their emotions, the more efficient and likely they are to produce their best performance [2]. In this regard, the effect of emotions on performance has been explored by coaches, sports managers, and athletes before, during, and following competitions [3]. Likewise, most athletes attribute their successful performance or performance leading to failure to a series of emotional factors [3].

Arousal must be kept at optimal levels in combat sports [4] to allow the athletes to react to suddenly arising opportunities in attack, as well as to avoid or block dangerous attacks from opponents [5]. Emotional intelligence (EI) is an essential component for combat sports practitioners, as they require maximum efforts related to mental stress and physical exhaustion, and a lack of EI can affect many performance dimensions [6].

Successful athletes have been shown to have special abilities and talents due to their EI [7]. EI is a set of non-cognitive abilities and skills that successfully affect coping with environmental demands, including tournament requirements and pressures [8]. Athletes with higher levels of EI perform better in team sports such as cricket [9], hockey [10], or baseball [11], with significant positive correlations observed between EI and athletic performance. In addition, EI is advantageous for psychological skills and factors that transcend sport [12]. For example, in a study investigating the correlations between EI and athletic performance (draft rank, total games played, years since draft, and total points), Perlini & Halverson surveyed 76 hockey athletes. The results showed that players who scored higher on average EI scored higher on interpersonal relationships, stress management, self-consciousness, emotional management, and stress tolerance [10]. In addition, athletes with open skills (footballers) were better at using EI than those with closed skills (gymnasts), resulting in better athletic performances.

Mental toughness is defined as the “ability to withstand pressures and hardships, overcome obstacles and failures, focus on performance goals, keep calm after failure and consistently perform at high levels of competition and competitiveness” [13]. Furthermore, it has been reported that mental toughness is the most important feature for determining performance success in athletes [14, 15]. Mental toughness characteristics prepare athletes and make them succeed in challenging sporting environments, stressful training, competition, and post-competition situations. Hence, managers, athletes, coaches, and psychologists see mental toughness as a method to achieve success, overcome failures, and develop mental skills [13, 16]. Jones et al. interviewed elite and super-elite athletes from various sports. They concluded that toughness includes a developed or innate (genetic) psychological boundary that enables the individual to better cope with the needs that emerge from exercise (competition and training) and lifestyles. This ability helps athletes to focus during competition against opponents, exhibiting determination, focus, self-confidence, and controlling stressful situations [17].

Mindfulness modulates emotions without judgment and increases consciousness of mental and physical emotions, helping recognise and accept emotions and physical phenomena clearly, as they occur [18, 19]. Furthermore, the consciousness of the moment also helps athletes understand and manage motivation levels in a self-efficient way [20], thereby improving athletic performance. Furthermore, a high level of mindfulness allows athletes to focus more on competition and gives them a sense of control, leading to improved skilful performance-related outcomes [21, 22]. Therefore, psychological skills, including EI, mental toughness, and mindfulness, improve the athlete’s performance and superiority in competitions [23].

However, to the best of our knowledge, even though an accumulating body of scholarly research has shown a positive and significant relationship between EI and sports performance [11, 24–28], no previous studies have examined the relationship between EI, mental toughness, and mindfulness, anaerobic and aerobic fitness in elite taekwondo athletes. Therefore, the purpose of this study was to investigate the relationship between EI, mental toughness and mindfulness, anaerobic and aerobic performance in a group of elite taekwondo athletes. Our study assumptions were that mental skills and physiological abilities are interrelated in elite taekwondo athletes.

## 2. Materials and methods

### 2.1. Design and subjects

This cross-sectional study comprised ten elite Iranian male taekwondo athletes who were members of the Iranian national team. All subjects actively participated in the Iranian Taekwondo Adult Premier League (2018–2019) before the COVID-19 pandemic. Inclusion criteria were that athletes needed to be members of the national team and/or compete in the Asian Championships, Asian Games, or international tournaments between 2017–2018. Participants had 13 ±3 years of training experience and served 4 ±1 years as national team members. All athletes weighed in during the League for their teams to compete in weight categories ranging from -58 kg to -74 kg. Due to competition in the aforementioned Premier League, participants were involved in weight loss procedures, with minimal weight loss between 1 and 2.5 kg occurring over a monthly period before and during the study. Exclusion criteria included serious medical conditions, consumption of illegal substances, or musculoskeletal injuries. The criteria also included refraining from alcohol intake for at least one week before the study. All subjects read and signed informed consent forms before participating in the study. They were also informed that they could withdraw from the study at any time. This study was ethically approved by the Sport Science Research Institute of Iran (IR.SSRC.REC.1399.062), and was conducted following the Declaration of Helsinki regarding human research protection.

### 2.2. Measures

All the participants completed four questionnaires under the same environmental conditions (i.e., quiet room, temperature, light, etc.). The same-trained researcher supervised the completion of all the measurements.

#### 2.2.1. Emotional intelligence scale

The “Emotional Intelligence Scale” (EIS) was initially developed by Schutte et al. [29], following the model by Salovey and Mayer [30] and subsequently validated by Lane et al. [31, 32]. In the sports arena, EIS is a self-assessment scale questionnaire consisting of 33 items, three of which 5, 28 and 33 are reverse-scored, and six factors that show the individual’s desire and persistence to continue participating in sports activities. The six factors in this questionnaire include: I) evaluating other people’s feelings (seven items), II) evaluating one’s feelings (five items), III) self-regulation (five items), IV) social skills (five items), V) using emotions (seven items), and VI) optimism (four items). Items are presented in the form of declarative sentences and questions, and each is answered on a five-point Likert scale, ranging from strongly disagree-1 to strongly agree-5, and scored as 2 = disagree, 3 = neutral, and 4 = agree [29]. The average score can range from 33 to 165, with higher scores indicating more characteristics of emotional intelligence. The mean emotional intelligence score is approximately 124 (with a standard deviation of about 13), with scores <111 or >137 being considered unusually low or high [33].

Lane et al. [32] provided a comprehensive overview of the questionnaire’s development, using two studies to present its content validity and confirmatory factor analysis (CFA) of the subscales. In addition, Eydi et al. [34] provided evidence of its factor analysis and reliability in a translated version for those who speak Farsi. In the present study, CFA could not be calculated due to small sample sizes; however, Chronbach’s alpha coefficients ranged between 0.75 and 0.87 across subscales and the coefficient was 0.80 overall. Although Lane et al. [2] suggested removing 14 items (like “I find it hard to understand the non-verbal messages of other people” and “When I am faced with obstacles, I remember times I faced similar obstacles and overcame them”) that lacked emotional content, since Eydi et al. [34] validated the original 33-item version, this was employed in the present investigation.

#### 2.2.2. Sport Success Scale (SSS)

The “Sport Success Scale” (SSS) was designed based on a robust conceptual framework devised by drawing from the theory of dynamical systems, the multi-stage theory of motor learning, and the motivation theory, and standardised by Mousavi and VaezMousavi [35]. This tool consists of 29 questions that measure flow state, attention, technique, sensitivity to error, commitment, and achievement. This scale is set on a six-point Likert scale, ranging from strongly disagree-1 to strongly agree-6. Reliability (Cronbach’s alpha coefficients) was estimated as follows: flow state 0.89, attention 0.88, technique 0.89, sensitivity to error 0.88, commitment 0.89, achievement 0.89 and overall alpha coefficient 0.89. In addition, the content-related validity and structure-related validity were confirmed to be satisfactory [35].

#### 2.2.3. Sport Mental Toughness Questionnaire (SMTQ)

This questionnaire has been designed to measure mental toughness with three subscales: confidence, constancy, and control [36, 37]. The SMTQ includes 14 questions that measure three subscales: confidence (six questions), constancy (four questions), and control (four questions). Each question has four answer options, from completely incorrect and almost incorrect to almost and completely correct. Details on score calculations are provided elsewhere [36, 37]. The validity and reliability of this questionnaire have been confirmed by Sheard and collaborators [37]. A Cronbach’s alpha coefficient of confidence, constancy, and control was reported to be 0.80, 0.74, and 0.71, respectively [37]. The SMTQ is the only valid and reliable tool in the field of mental toughness that includes emotion control and negative energy, which have been identified as critical characteristics of successful athletes [17, 38–40].

#### 2.2.4 Mindfulness Inventory for Sport (MIS)

This inventory consists of 15 questions and uses three factors of awareness (five questions), non-judgment (five questions), and refocus (five questions) to evaluate the athlete’s mindfulness [41]. The sum of these three dimensions determines the overall score of sport mindfulness. Each question has six options: not at all, very little, to some extent, a lot, very much, and always, scoring one to six for each option. Therefore, the minimum score is 15, and the maximum is 60 [41].

#### 2.2.5. Morphological assessment

A calibrated digital scale (SECA 803, Hamburg, Germany) was used to measure body weight with an accuracy of 0.1 kg. Body height was assessed using a stadiometer (SECA 206, Hamburg, Germany) with an accuracy of ±1cm. Body mass index (BMI, kg/m^2^) was calculated using weight (kg) divided by the square of height (m). An International Society for the Advancement of Kinanthropometry (ISAK) level 1 accredited anthropometrist (technical error of measurement (TEM) of 2.5% for skinfolds) performed all skinfold measurements in line with standardised ISAK procedures [42]. 8-site skinfold thickness according to ISAK; (biceps, triceps, subscapular, iliac crest, supraspinal, abdominal, anterior thigh, and medial calf) were measured with a caliper (Slimguide, Health Products, Plymouth, USA). All measures were recorded twice on the right-hand side of the participant’s body. A third measurement was taken if the difference between duplicate measures exceeded 5% for skinfolds. The mean of duplicate or median of triplicate measurements was used for all subsequent analyses [43, 44]. The skinfold thickness values were then used to calculate body density (BD) by utilising Jackson and Pollock’s method and body fat percentage (%BF) was calculated by using Brozek’s formula [45].

#### 2.2.6. Anaerobic and aerobic fitness

To determine anaerobic and aerobic fitness, the Wingate (WAnT) Ergomedic Cycle (Monark, E894) and the Bruce test on a calibrated treadmill (Rodby^™^, RL 1600E, Enhorna, Sweden) were used, respectively. Before data collection, the test procedures were explained to the subjects and all were fully familiarised with testing procedures. The assessments were performed on a single day between 1:00 and 5:00 pm. This timing was observed to control for diurnal variation as well as to replicate tournament fighting times and, therefore, was tournament specific. A rest interval of 3 hours between each test was granted to ensure adequate recovery, restoration of muscle glycogen, and to prevent injury. At arrival, participants completed a 15-minute standardised warm-up. The WAnT test was performed first. During the test, participants performed a maximal 30-second trial with the resistance load set at 7.5% of the subject’s body weight [46]. Variables for peak power and relative peak power to body mass were reported and recorded for further analysis.

It was reported that the recovery time of only 20 minutes is long enough for sufficient recovery after a specific high-intensity test such as WAnT to achieve max Vo_2max_ criteria in an anaerobic test performed on the same day [47]. Therefore, a 3-hour break was determined to be a sufficient period to ensure adequate recovery of athletes between tests [48]. During the recovery, the consumption of carbohydrate sports drinks and inactive rest were observed. Following the 3h break, the Bruce test was administered under the supervision of a trained exercise physiologist. Prior to the start, participants performed a 15-minute standardised warm-up. During the Bruce protocol, participants started at a calibrated treadmill (Rodby^™^, RL 1600E, Enhorna, Sweden) speed of 2.7 km. h-1 with an inclination of 10% gradient for 3 minutes. Workloads (speed and inclination) were simultaneously increased every 3 minutes until volitional exhaustion was reached, as previously outlined [49]. The final time was recorded when the subject was unable to continue. VO2 peak was calculated as outlined in a previous study [49]. Maximum heart rate (HR -MAX) was achieved via an aerobic test on a treadmill which was monitored via a chest belt (Polar H10, Kempele, Finland).

### 2.3 Statistical analysis

Descriptive statistics with mean, standard deviation (SD) and 95% confidence intervals were used to describe central tendencies and variability. The Shapiro-Wilk and Levene’s tests were used to evaluate the normality of data distribution and analyse the homogeneity of variance, respectively. The Spearman rank Correlation Coefficient was utilised to examine the relationship between the subscales of EI, sports success, mental toughness and mindfulness and anaerobic and aerobic performance. Data were processed and analysed using the SPSS for Windows (Version 22.0; SPSS, Inc., Chicago, IL, USA). Statistical significance for all tests was set at p ≤ 0.05

## 3. Results

The subjects had a mean age of 20.6±2 years, height of 180.90±1.73 [95%CI 179.66 to 182.14] cm, weight of 61.50±1.65 [95%CI 60.32 to 62.68] kg, BMI of 18.78±0.62 [95%CI 18.34 to 19.22] kg/m^2^, and fat percentage of 8.87±1.46 [95%CI 7.83 to 9.92]. Descriptive statistics, including mean and standard deviation related to emotional intelligence, sports success, mental toughness, mindfulness, Bruce & Wingate tests, and their calculated subscales, are presented in Tables 1–5.

**Table 1:** Mean and standard deviation for emotional intelligence and its subscales in Iranian elite taekwondo athletes.

SEIQ Subscales	Mean ± SD	95% CI	
		Lower	Upper
**Evaluation Others`Feelings**	20.60 ± 2.91	18.52	22.68
**Evaluation Own Feelings**	19.00 ± 4.71	15.63	22.37
**Self-Regulation**	18.40 ± 4.48	15.20	21.60
**Social Skills**	18.10 ± 4.18	15.11	21.09
**Employing Emotion**	28.30 ± 6.63	23.55	33.05
**Optimism**	15.30 ± 3.80	12.58	18.02
**Emotional Intelligence**	119.90 ± 24.64	102.27	137.53

**Table 2:** Mean and standard deviation for sport success and its subscales in Iranian elite taekwondo athletes.

Subscale	Mean ± SD	95% CI	
		Lower	Upper
**Flow state**	25.40 ± 5.17	21.70	29.10
**Attention**	25.40 ± 3.81	22.68	28.12
**Technique**	21.80 ± 2.70	19.87	23.73
**Sensitivity to Error**	26.90 ± 2.81	24.89	28.91
**Commitment**	27.00 ± 3.86	24.24	29.76
**Achievement**	27.40 ± 4.01	24.54	30.27
**Sport Success**	180.40 ± 23.86	163.33	197.47

**Table 3:** Mean and standard deviation for mental toughness and its subscales in Iranian elite taekwondo athletes.

Subscale	Mean ± SD	95% CI	
		Lower	Upper
**Confidence**	15.40 ± 3.20	13.11	17.69
**Control**	10.50 ± 1.84	9.18	11.82
**Constancy**	13.20 ± 4.16	10.23	16.17
**Mental Toughness**	39.40 ± 7.15	34.28	44.52

**Table 4:** Mean and standard deviation for mindfulness and its subscales in Iranian elite taekwondo athletes.

Subscale	Mean ± SD	95% CI	
		Lower	Upper
**Awareness**	21.10 ± 4.84	17.64	24.56
**Non-judgmental**	17.10 ± 2.69	15.18	19.02
**Refocusing**	20.90 ± 4.15	17.93	23.87
**Mindfulness**	58.80 ± 7.61	53.35	64.25

**Table 5:** Mean and standard deviation for Bruce & Wingate tests.

Subscale	Mean ± SD	95% CI	
		Lower	Upper
**Wingate test**			
**PP (w)**	572.10 ± 8.31	566.16	578.04
**RPP (w/kg)**	11.84 ± 1.11	11.05	12.63
**Bruce test**			
**VO**_**2peak**_ **(L/min)**	3.50 ± 0.28	3.30	3.70
**VO**_**2peak**_ **(ml/kg/min)**	57.00 ± 8.83	50.68	63.32
**HR-MAX (bpm)**	202 ± 2.0	200	203

**PP**: peak power; **RPP**: relative peak power; **HR-MAX**: maximum heart rate

The Spearman rank correlation coefficients are presented in Table 6. Statistically significant correlations were noticed between the evaluation of feelings (EI scale) and VO_2_peak (ml/kg/min) (r = -0.70, p = 0.0235); between social skills (EI scale) and relative peak power (w/kg) (r = 0.84, p = 0.0026); between optimism (EI scale) and VO_2_peak (ml/kg/min) (r = -0.70, p = 0.0252) and between optimism (EI scale) and HR-MAX (r = -0.75, p = 0.0123); and, finally, between control mental toughness scale) and relative peak power (w/kg) (r = 0.67, p = 0.0360).

**Table 6:** Correlations between emotional intelligence, sports success, mental toughness and mindfulness, aerobic and anaerobic fitness.

Scale/sub-scale	Peak power (W)	Relative peak power (W/kg)	VO_2_peak (L/min)	VO_2_peak (ml/kg/min)	HR MAX (bpm)
Evaluation of others’ feelings	0.26 0.4695	0.17 0.6464	0.34 0.3324	-0.28 0.4280	-0.28 0.4388
Evaluation of own feelings	0.47 0.1686	0.16 0.6527	0.55 0.1025	-0.70* 0.0235	-0.55 0.1034
Self-regulation	-0.56 0.0948	-0.46 0.1843	-0.56 0.0910	-0.25 0.4896	-0.29 0.4216
Social-skills	0.41 0.2437	0.84* **0.0026**	0.37 0.2900	0.10 0.7941	0.19 0.5909
Employing emotions	-0.34 0.3324	-0.16 0.6684	-0.38 0.2738	-0.18 0.6185	-0.01 0.9722
Optimism	0.42 0.2317	0.20 0.5868	0.48 0.1563	-0.70* **0.0252**	-0.75* 0.0123
Emotional intelligence	0.36 0.3094	0.18 0.6185	0.45 0.1923	-0.435 0.2092	-0.425 0.2203
Flow state	-0.32 0.3611	0.12 0.7454	-0.43 0.2176	0.317 0.3725	0.343 0.3321
Attention	-0.16 0.6611	-0.12 0.7454	-0.20 0.5832	-0.15 0.6810	0.11 0.7666
Technique	-0.20 0.5832	0.07 0.8498	-0.23 0.5194	-0.36 0.3015	-0.25 0.4860
Sensitivity to error	-0.46 0.1866	-0.33 0.3529	-0.52 0.1217	0.21 0.5581	0.33 0.3515
Commitment	-0.19 0.6016	-0.01 0.9726	-0.24 0.5019	-0.23 0.5306	-0.02 0.9579
Achievement	-0.10 0.7941	0.27 0.4592	-0.17 0.6336	-0.14 0.7020	0.07 0.8595
Sport success	-0.41 0.2364	-0.25 0.4833	-0.47 0.1683	-0.01 0.9866	0.18 0.6275
Confidence	-0.28 0.4342	-0.15 0.6881	-0.32 0.3665	-0.31 0.3829	-0.19 0.6035
Control	0.47 0.1733	0.67* **0.0360**	0.44 0.2029	-0.24 0.5115	-0.22 0.5372
Constancy	-0.54 0.1059	-0.24 0.5056	-0.63 0.0522	0.15 0.6723	0.25 0.4845
Mental toughness	-0.36 0.3113	-0.14 0.6974	-0.42 0.2316	-0.22 0.5514	-0.09 0.8094
Awareness	-0.32 0.3658	-0.30 0.4073	-0.29 0.4220	-0.33 0.3482	-0.30 0.3965
Non-judgmental	0.19 0.6016	0.57 0.0849	0.10 0.7800	-0.24 0.5073	-0.07 0.8465
Refocusing	0.24 0.5101	0.55 0.0974	0.21 0.5650	-0.09 0.7979	-0.22 0.5492
Mindfulness	0.15 0.6865	0.18 0.6185	0.13 0.7226	-0.09 0.8112	-0.20 0.5734

Note: The first line presents *r* and the second line presents the *p*-value of correlations statistics;

*—statistically significant results p ≤ 0.05

## 4. Discussion

The present study investigated the relationships between mental skills and anaerobic and aerobic fitness in elite taekwondo athletes. The results showed a significant relationship between some subscales of the EI scale and performance outcomes. The ability to withstand pressures and hardships, overcome obstacles and failures, focus on goals, keep calm after failure, and consistently perform at high levels of competition and competitiveness are all defined as mental toughness attributes of paramount importance to athletes. This makes athletes successful in difficult, stressful situations such as training, competition, and post-competition [14].

To date, very few studies have investigated psychological skills and relationships with anaerobic and aerobic performance in taekwondo practitioners. More importantly, no research exists on psychological skills and elite taekwondo athletes. Interviewing hundreds of athletes, Loehr found striking similarities in the athletes’ experiences that led to their high performance and estimated that approximately 50% of sports successes were due to psychological factors, namely mental toughness. He discovered that toughness allows a person to demonstrate their skills and talents despite intense pressure [40]. Research findings from the World Cup and Olympic championship interviews confirmed these mental toughness definitions [39, 50, 51]. Accordingly, the results also showed a significant relationship between the subscale of control (mental toughness scale) and relative peak power. Furthermore, a high level of mental toughness allows athletes to focus more on competition and provides a sense of control which could lead athletes to focus better on the performance tasks. Additionally, it was reported that a greater focus and control over competition also leads to improved skill performance [21] and, in our case, to a greater anaerobic output. Therefore, it can be stated that athletes with more mental toughness and acceptance and less repression of thoughts are less reactive and respond much better to factors such as leaving a training program, which may affect the initiation and adherence to exercise. In this regard, Golby and Sheard examined 114 rugby players at three different levels of competition: International, Premier League, and regional. They found that athletes who competed at the highest level had significantly higher mental toughness than athletes who competed at lower levels [37]. However, similar to our study results, associations between subscale control (mental toughness scale) and relative peak power were noted in the aforementioned study. Additionally, these findings demonstrate that we cannot attribute these subscales to other variables in elite taekwondo athletes.

Despite all these factors, an athlete who can manage their emotions, take their motivation out of a passive state, and use the situation to achieve the desired result with skills such as optimism and self-confidence would have better chances of success. Our findings indicate significant correlations between the evaluation of own feelings with peak power and optimism with peak power and HR MAX. Previous research has shown that good psychological states can contribute to better anaerobic performance and, consequently, a higher HR [52], which could explain the present study’s associations between these variables. These variables-subscales are related to the EI scale linked to an athlete’s performance. In addition, it was reported that the lack of cognition and control of emotions could negatively affect the athlete’s other abilities, such as composure, concentration, and game planning [31], directly impacting sports performance.

Barlow has previously stated that reducing anxiety is one of the beneficial aspects of EI and helps individuals skillfully manage stress [27]. Their findings showed that athletes’ cortisol levels rise under intense pressure and harsh conditions [27]. Elevated cortisol levels can have a negative effect on an individual’s performance [53]. In general, consciousness and the ability to understand the emotions of others provides individuals with the strength to achieve goals successfully, and coaches and players can create a team spirit and an effective sports environment. Also, athletes with high EI are more effective in revealing their abilities and applying them to positive actions. They also recognise their emotional abilities, strengths, and weaknesses. These skills determine their behaviour and performance [31]. Therefore, athletes can use the components of EI as a strategy to increase performance and control emotions effectively. Laborde’s meta-analysis stated that successful performance depends on emotions, physiological responses to stress, and psychological skills [28].

In general, the strongest athletes need EI training. This need does not stem from psychological problems; for better communication between body and mind and more effective performance, this is something they must develop. The sum of these skills and mental abilities indicates that athletes have more control over challenging and tense competition conditions than their opponents, and by maintaining focus and belief in their abilities to turn these challenges into opportunities, consistency and better concentration in performance.

There are some limitations to note in the present study. Due to the athletes’ involvement in competitions, the coaches’ fear of injury, and their time availability for testing, our testing battery was limited. However, the athletes measured were elite performers; therefore, the measurements obtained were valuable in determining key performance indicators in an elite group. The aforementioned prevented us from recruiting more study subjects to reach a larger sample size and to perform more tests. Additionally, direct aerobic measurement using gas analysers could provide more accurate measurements of aerobic capacity, and a specific taekwondo test could provide additional athletic information. Nonetheless, all the subjects tested were elite performers and medalists in international competitions. Moreover, the fact that participants were involved in regular training, competitions and weight loss allowed us to obtain the measurements of the athletes in a realistic sports environment. This adds to the applicability of current study findings. For future studies, it may be better to design data collection periods structured for inclusion during athletes’ attendance at training camps that are organised before competitions, which could lead to greater sample sizes and ensure that athletes are in top physical condition. Further studies should also focus on comparing men and women based on weight categories with larger sample sizes so that the results observed in the current study can be cross-validated.

The findings presented here indicate that elite taekwondo athletes need high levels of psychological skills, and these skills are related to fitness levels. Furthermore, the findings suggest that for future preparation for competitions, the athletes should be in their peak physiological and psychological form before every competitive event to increase their chances of success. By achieving optimal readiness, they can compete with high levels of self-confidence and self-regulation. Additionally, psychological tests used in the present study could be used to test and enhance the aforementioned skills, while our results could be used as normative values for elite taekwondo athletes. Therefore, psychological training would also be recommended in elite taekwondo athletes before high-level competitions in order to increase success rates [54, 55]. However, further studies are needed to explore these practical suggestions in elite taekwondo athletes.

## 5. Conclusions

Physical fitness is seen as a prerequisite for improving performance and sporting success. The present study showed a strong association between psychological factors and anaerobic and aerobic fitness. Furthermore, improving physical fitness is correlated with improvement in mental abilities, which indicates an ability to provide greater technical execution in minimum time in most competitions. Overall, the findings indicate that highly talented taekwondo athletes at the elite level with strong mental abilities appear to develop their physical fitness and psychological abilities to a greater extent than athletes at lower levels of performance.

## Supporting information

S1 Dataset (XLSX)
